# PD01 Budget Impact Analysis Of Expanding Newborn Inherited Metabolic Diseases Screening In Shanghai, China

**DOI:** 10.1017/S026646232400271X

**Published:** 2025-01-07

**Authors:** Dunming Xiao, Shanyan Zhou, Yingyao Chen

## Abstract

**Introduction:**

China has a high incidence of birth defects. Tandem mass spectrometry (MS/MS) screening enables rapid detection of multiple inherited metabolic disorders and has been widely promoted globally. This study aimed to conduct a budget impact analysis of replacing phenylketonuria screening with MS/MS by calculating the financial impact of reimbursing the costs of MS/MS screening.

**Methods:**

An Excel-based budget impact analysis model for MS/MS screening was developed. The number of newborns in Shanghai from 2024 to 2026 was estimated using the birth rate trend among the permanent population of Shanghai over the past decade. By integrating clinical screening data, along with the corresponding screening costs and diagnostic fees for the gold standard test, the financial impact of replacing phenylketonuria screening with MS/MS screening was calculated. The screening data for this study was extracted from a tertiary hospital in Shanghai. Demographic data were obtained from statistical websites, while cost data were derived from literature and a tertiary hospital in Shanghai.

**Results:**

The fiscal expenditures for phenylketonuria screening were CNY1.75 million (USD0.25 million), CNY1.65 million (USD0.23 million), and CNY1.56 million (USD0.22 million) for 2024, 2025, and 2026, respectively. In contrast, the corresponding fiscal expenditures for MS/MS were CNY25.23 million (USD3.54 million), CNY23.78 million (USD3.33 million), and CNY22.41 million (USD3.14 million). The additional fiscal expenditure for MS/MS, compared with phenylketonuria screening, was CNY23.48 million (USD3.29 million), CNY22.13 million (USD3.10 million), and CNY20.85 million (USD2.92 million), showing a yearly decreasing trend.

**Conclusions:**

The financial impact of MS/MS screening was controllable. It was recommended that the cost of MS/MS screening in Shanghai be covered by government funding. The promotion of newborn screening using MS/MS deserves priority consideration and publicity in Shanghai, China.

